# Association between relapses, stress, and depression in people with multiple sclerosis during the COVID-19 pandemic

**DOI:** 10.1007/s10072-022-05917-z

**Published:** 2022-01-29

**Authors:** Maddalena Sparaco, Giuseppina Miele, Luigi Lavorgna, Gianmarco Abbadessa, Simona Bonavita

**Affiliations:** Department of Advanced Medical and Surgical Sciences, University of Campania Luigi Vanvitelli, Piazza Miraglia, 2, 80138 Naples, Italy

**Keywords:** COVID-19, Multiple sclerosis, Relapse, Stress, Depression

## Abstract

**Background:**

Stress is a potential trigger for clinical and radiological activity in Multiple Sclerosis (MS). COVID-19 pandemic has been a relevant source of mental distress in people with MS (pwMS) and deeply impacted on disease management.

**Objective:**

To investigate the association between stress, anxiety, depression, and risk of relapse during the COVID-19 pandemic.

**Methods:**

From an electronic database used for clinical practice, we extracted data of relapsing–remitting (RR) or relapsing-progressive (RP) MS patients and calculated the annualized relapse rate (ARR) during 2019 and 2020. From 01/12/2020 to 30/12/2020, enrolled patients were invited to fill in a Google Forms survey to investigate depression, anxiety, stress, and Post-Traumatic Stress Disorder (PTSD).

**Results:**

We selected 216 patients with RR or RP-MS to calculate ARR: compared to 2019, in 2020 there was a significant increase in ARR (p = 0.0142).

Over 216 selected pwMS, 154 completed the survey. Matching the survey responses and incidence of relapses in 2020, there was a significant association between relapses and stress (p = 0.030**)** and relapses and depression (p = 0.011**)**, but not between relapses and anxiety (p = 0.130) or PTSD (p = 0.279).

**Conclusions:**

Our results support the hypothesis that pandemic-related stress is associated to clinical exacerbations, both as a possible consequence of the COVID-19 impact on MS care.

## Introduction

The role of stress in the pathogenesis of Multiple Sclerosis (MS) was initially highlighted by Charcot, who proposed that stress may trigger disease activity [1]. People with MS (pwMS) have long reported that psychological stress can worsen their symptoms; indeed, it has been demonstrated that exposure to a wide range of challenging life events is correlated with the worsening of neurological symptoms [2], increased lesion burden on the brain magnetic resonance imaging (MRI) [3], and relapses [4].

Mood disturbances are also very common among pwMS, with a higher prevalence of depression and anxiety compared to the general population [5]. Depression in MS has been widely investigated and, as supported by MRI studies [5], it is considered to be dependent on structural and functional damage due to MS pathology and not only reactive to a disabling disease.

On January 30th, 2020, the World Health Organization (WHO) declared Public Health Emergency for Novel Coronavirus disease (COVID-19) [6].

After 1 year of the COVID-19 pandemic, it is clear that it may cause mental health problems such as stress, anxiety, and depressive symptoms either in the general population [7] or in fragile patients such as pwMS [8, 9].

On a large MS sample recruited online during the first Italian lockdown, a higher proportion of pwMS were depressed, had a high level of perceived stress and felt significantly less social support compared to the general population [8].

Furthermore, standard of care for pwMS has been deeply disrupted by COVID-19–related restrictions, imposing limited access to in-person visits, MRI and laboratory tests, changes in the management of relapses, in the use of disease modifying therapies (DMTs), in the access to rehabilitation facilities, and to psychological support programs [10].

Given the premise that exposure to challenging life events may be correlated with higher disease activity in pwMS [3, 4], and the highly negative impact of COVID-19 on MS care, our study aimed to investigate the association between stress, anxiety, depression, and risk of relapse during the ongoing pandemic.

In particular, we investigated outpatient pwMS to evaluate whether in 2020 there was an increase of the annualized relapse rate (ARR) compared to 2019 and whether there was an association between the risk of relapse and stress, anxiety, and depression.

## Methods

This is a retrospective observational study involving pwMS afferent to the MS Center of the II Clinic of Neurology of the University of Campania “Luigi Vanvitelli”, Italy. The observation period was from January 1^st^, 2019 to December 31^st^, 2020.

Key eligibility criteria were: having a diagnosis of relapsing–remitting (RR) or relapsing-progressive (RP) MS according to the 2013 Lublin et al. criteria [11] and age > 18 years.

Exclusion criteria were: a diagnosis of MS in 2019 or 2020; progressive forms of MS without clinical activity, a COVID-19 diagnosis or positive serological test for SARS-CoV-2 infection, modification in disease-modifying therapy (DMT) schedule, and clinically relevant cognitive impairment during the observation period; further exclusion criteria, to minimize the influence of confounding factors potentially influencing the occurrence of MS relapses [12] were: pregnancy or breastfeeding, any infection occurring 5 weeks before and 2 weeks after the onset of a clinical relapse [13], and DMT switch in the 6 months before the onset of relapse.

Relapses were defined as a neurologic deficit lasting at least 24 h in the absence of fever or infections [14]. To exclude pseudo-relapses, we considered true relapses only those objectively evaluated with an in-person visit (in 2019 and 2020) or a tele-visit (exclusively in 2020) and treated with high dose steroids.

Referring to the observation study period, data of pwMS matching the inclusion criteria were collected from an electronic database used for clinical practice. In our MS Center, from 2017 pwMS are monthly contacted via Whatts App and invited to fill in a Google Forms with a diary of infections therefore data on the infections during the study period were collected from the excel files exported from the Google Forms diary.

Data collected from the clinical database included: age, sex, number of relapses, disease phenotype, disease duration, ongoing DMT.

To investigate depression, anxiety, stress, and the occurrence of Post-Traumatic Stress Disorder (PTSD), from 01/12/2020 to 30/12/2020, pwMS selected based on the inclusion criteria were invited by e-mail to link to a Google Forms survey.

The survey was composed by.

- A general part with questions relating to socio-demographic data (type of job, switch to smart working during the pandemic, relatives or friends diagnosed with COVID-19)
- A clinical part including:
  - Questions about autonomous changes in treatment schedule during the pandemic (March–December 2020)
  - The Italian version of the Patient Determined Disease Steps (PDDS) [15] for disability evaluation
  - The Short Screening Scale for DSM-IV (SSS DSM-IV) [16], to assess the presence of PTSD symptoms (cut off score ≥ 4).
  - Depression, Anxiety and Stress Scale (DASS-21) [17], to assess the presence and levels of Depression, Anxiety, and Stress with 7 items for each mood disturbance.

Levels of depression, anxiety, and stress were identified according to the scores for each scale, as below:

**Table:** 

	Depression	Anxiety	Stress
Normal	0–4	0–3	0–7
Mild	5–6	4–5	8–9
Moderate	7–10	6–7	10–12
Severe	11–13	8–9	13–16
Extremely severe	> 14	> 10	> 17

Data collected from the clinical database and survey were matched and used for statistical analysis.

The Institutional Review Board of the University of Campania ‘Luigi Vanvitelli’ has approved the study procedure in the form of a retrospective observational study and web survey. The Legislative Decree n. 101/2018 Code regarding the protection of personal data was followed. The enrolled subjects consented to the use of recorded surveys for scientific purposes on aggregate level and agreed to the data processing at the beginning of the survey where a clear explanation about the personal data that would have been collected and what used for was given. Furthermore, no electronic ‘cookies’ were embedded and encryption was applied to allow only approved users to access the full data set.

### Statistical analysis

The categorical variables were reported as number and percentage (%), while the continuous ones were expressed as mean ± standard deviation (SD) and min and maximum. We categorized mood disorders in mild, moderate, severe, and extremely severe, as previously described.

Wilcoxon test was used to examine the difference in ARR between 2019 and 2020. McNemar test was used to compare the frequencies of pwMS treated with II line DMTs in 2019 and 2020.

“U Mann-Whitney and chi-square tests were used to compare clinical and demographic characteristics between dropped-out and included patients.”

Sensitivity analysis excluding patients with more than one relapse was performed to verify whether data might have been driven by outliers.

Logistic regression was used to investigate the risk of relapse and to examine the association with stress, anxiety, and depression in 2020. The regression models were adjusted for age, sex, disease duration, disability level, treatment, and disease course. Odds ratio (OR) and confidence interval (CI, 2.5–97.5%) were calculated. The level of significance was set at 0.05. All analyses were performed using R software (Version 1.4.1106).

## Results

### Annualized relapse rate

From a cohort of 369 pwMS, we selected 216 relapsing (RR, RP) patients to calculate the ARR.

143 patients were excluded for being progressive without clinical activity.

One further patient was excluded for pregnancy in 2020 and another one for diagnosis in 2020; 5 patients were excluded in 2019 and 2 in 2020 for DMT switch in the 6 months before the onset of relapse: No patients were excluded for COVID-19 diagnosis or positive serological test for SARS-CoV-2, breastfeeding, any infection occurring 5 weeks before and 2 weeks after the onset of a clinical relapse. Only one patient was excluded because of a clinically relevant cognitive impairment during the observation period.

Fifty-three relapses were registered in 2019 and 76 in 2020.

According to the inclusion and exclusion criteria, in 2019 we excluded 7 relapses occurring during the DMT switching period (exclusion criteria); in 2020, we excluded 4 relapses during the DMT switching period (exclusion criteria), 1 occurring in pregnancy, and 1 because occurring in a patient diagnosed in 2020. Therefore, 46 relapses (in 46 pwMS) were included in 2019 and 70 (in 56 pwMS) in 2020. 67.3% of pwMS with relapses were treated with II line DMTs in 2019 and 71.4% in 2020 (p = 0.176).

ARR was 0.22 ± 0.42 in 2019 and 0.32 ± 0.60 in 2020. Compared to 2019, in 2020 there was a significant increase in the ARR (p = 0.0142).

**Fig. 1:**
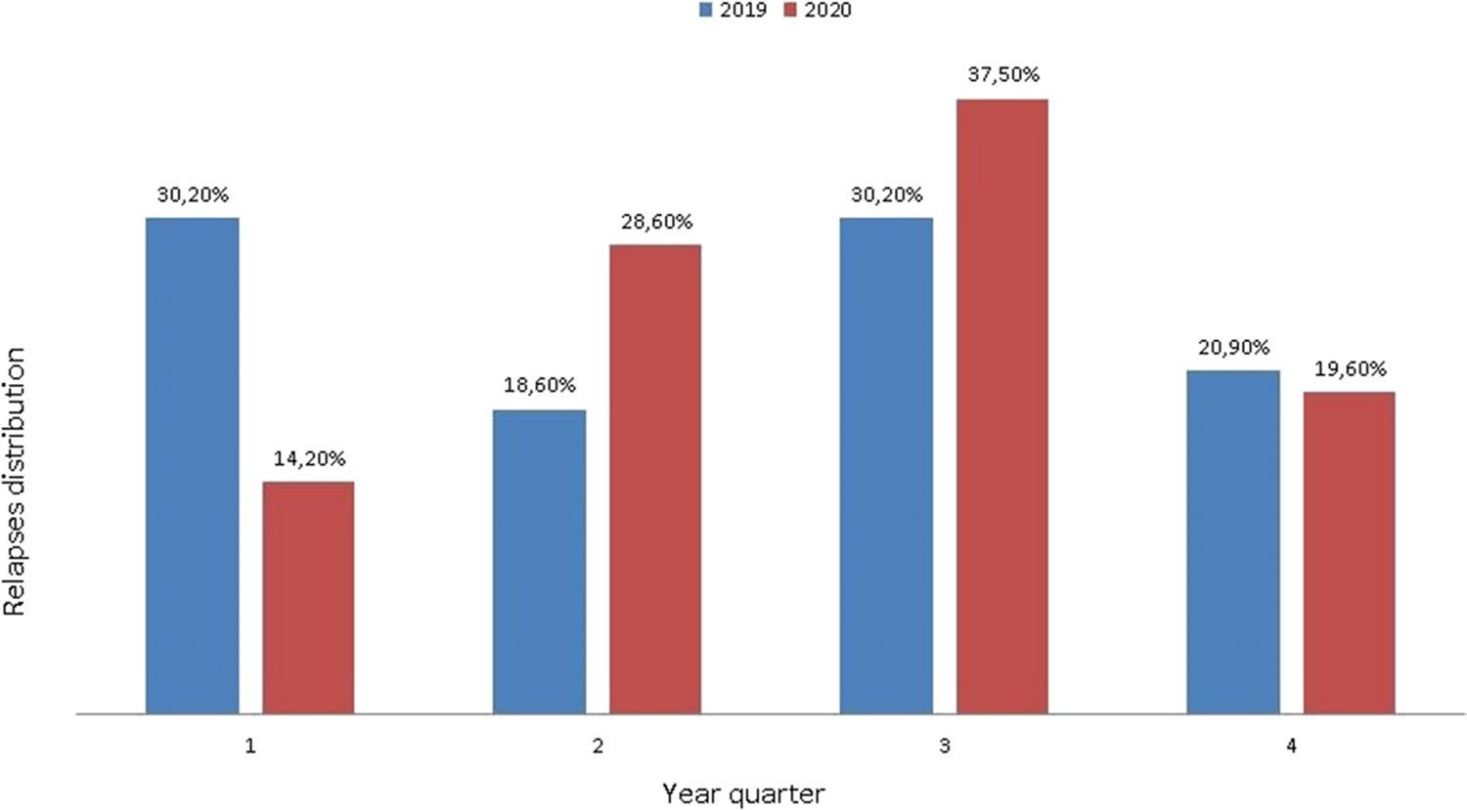
Distribution of relapses in 2019 and 2020 according to year’s quarters

Furthermore, comparing the distribution of relapses in 2019 and 2020, the increase in relapses was mainly in the second and third quarters of 2020, in the months following the declaration of the pandemic (see Fig. 1).

### Survey

We sent an e-mail with the survey link to the 216 pwMS selected according to the inclusion/exclusion criteria.

154 pwMS completed the survey (see Table 1 for demographic and clinical characteristics).

**Table 1:** Demographic and clinical characteristics of 154 people with MS (pwMS)

	Min	Max	Mean	SD	% (n)
Sex (female)					70.8% (109)
PwMS with relapse					30,5% (47)
Not employed subjects					9.09% (14)
Smartworking:					
- Never					67.5% (104)
- Only during March–May 2020					20.1% (31)
- Throughout 2020					12.3% (19)
Acquaintance with COVID-19					64.3%
Age	20	71	43,8	10,5	
Disease Duration	3	45	16,49	9,36	
Number of cohabiting			2,9	1,36	
PDDS score	0	7	1,74	1,74	
SSS DSM-IV score	0	7	2,81	2,09	
Stress (DASS-21)	0	21	9,39	5,79	
Anxiety (DASS-21)	0	21	5,72	5,36	
Depression (DASS-21)	0	21	7	6,05	

SD: standard deviation, n: number of subjects, PwMS: people with multiple sclerosis, SSS DSM-IV: Short Screening Scale for DSM-IV, PDDS: Patient-Determined Disease Steps

There were no significant differences in terms of demographic features between pwMS who completed (n. 154) and those who did not complete (n. 62) the survey (see Table 2). Considering only the 154 patients who completed the survey, we observed a lower ARR in 2019 compared to that observed in 2020 (0.21 vs 0.35, respectively. P value = 0.0174).

**Table 2:** Demographic and clinical data of dropped-out and included patients

	Dropped-out patients (n = 62)	Included patients (n = 154)	P value
Age (SD)	45.13 (10.34)	43.8 (10.5)	0.1781
Female sex (n/%)	46 (71.5%)	109 (70.8%)	0.302
ARR 2019 (SD)	0.225 (0.421)	0.209 (0.423)	0.7237
ARR 2020 (SD)	0.241 (0.563)	0.357 (0.612)	0.1359
DMTs -			0.705
(I line n/%)	19 (30.64%)	61 (39.61%)	
(II line n/%)	43 (69.36%)	93 (69.39%)	

The mean age of participants was 43.8 years ± 10.5, (range: 20–71); 70.8% (109) were females. Mean DD was 16.49 years ± 9.36. 130 were RR pwMS and 24 RP pwMS. Mean PDDS was 1.7 ± 1.74 (range: 0–7). 30.5% (47) of participants had at least one relapse in 2020.

9.1% (14) of subjects were not employed; 67.5% (104) of participants have never been in smart-working in 2020, 20.1% (31) were on smart working only during the first pandemic wave (March–May 2020), while 12.3% (19) have been in smart-working throughout all 2020.

Only 3 pwMS had no cohabiting; in the remaining pwMS, the mean of cohabiting was 2.9 ± 1.36. Finally, 64.3% of the participant had at least one acquaintance with the COVID-19 diagnosis.

The mean score for SSS DSM IV was 2.81 ± 2.09 (range: 0–7).

Considering SSS DSM-IV, 37% (57) pwMS have been diagnosed with PTSD (score ≥ 4).

The mean DASS-21 score was 9.39 ± 5.79 (range: 0–21) for stress, 5.72 ± 5.36 (range: 0–21) for anxiety, and 7 ± 6.05 (range: 0–21) for depression.

Considering DASS-21,

- 44.2% (68) pwMS had no stress, 11% (17) had mild level of stress, 12,3% (19) moderate, 15,6% (24) severe, 16.9% (26) extremely severe.
- 43.5% (67) pwMS had no axiety, 16.2% (25) mild level of anxiety, 6.5% (10) moderate, 8.4% (13) severe, 25.4% (39) extremely severe.
- 44.2% (68) pwMS had no depression, 9.7% (15) mild level of depression, 18.8% (29) moderate, 7.8% (12) severe, 19.5% (30) extremely severe.

**Fig. 2:**
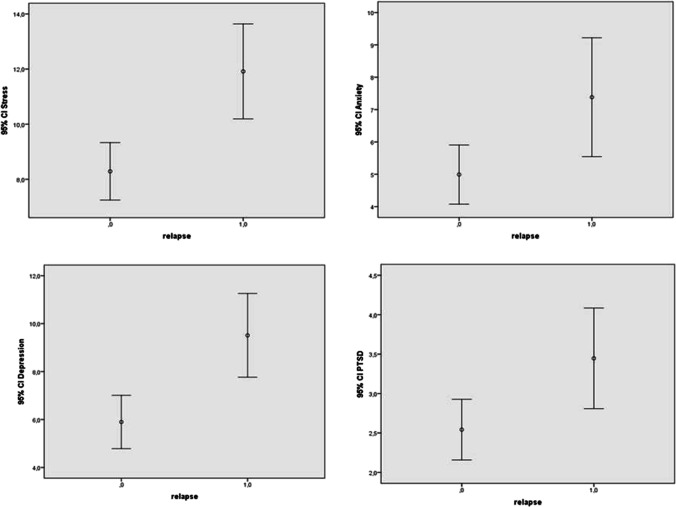
SSS DSM-IV and DASS-21 scores in relation to relapses. Box-plot shows means and standard deviations for each mood disorders

Figure 2 shows the mean score and SD at SSS DSM-IV and DASS-21 in relation to relapses.

We calculated the association between mood disorders and relapses in 2020 for the participants completing the survey.

There was a significant association between relapses and stress (p = 0.030**)** and relapses and depression (p = 0.011**)**, but not between relapses and anxiety (p = 0.130) or PTSD (p = 0.279) (see Table 3).

**Table 3:** Association between each mood disorder and relapses

	Odds Ratio	P value	CI 95%
Stress	1.087875	0.030*	1.008163—1.17389
Anxiety	1.059501	0.130	0.9831773—1.14175
Depression	1.09503	0.011*	1.020602—1.174886
Post-Traumatic Stress Disorder	1.113037	0.279	0.9169235–1.351096

CI: confidence interval; *significant

Regarding the class levels for stress, there was a significant association between relapses and extremely severe levels of stress (p = 0.025**)**, while, considering the class levels for depression, there was a significant association between relapses and moderate, severe, and extremely severe levels of depression (p = 0.036 for moderate, p = 0.046 for severe, p = 0.039 for extremely severe) (see Table 4).

**Table 4:** Association between each mood disorder (categorical) and relapses

	Odds Ratio	P value	CI 95%
Stress			
Mild	1.329983	0.694	0.3220401—5.492651
Moderate	2.383176	0.190	0.6500617—8.736905
Severe	1.679038	0.406	0.4943059—5.703285
Extremely severe	3.797583	0.025*	1.183694—12.18358
Anxiety			
Mild	2.719792	0.090	0.8564257—8.637371
Moderate	3.68394	0.105	0.7614411—17.82332
Severe	1.466254	0.633	0.3049545—7.049902
Extremely severe	1.881798	0.236	0.6608215—5.358731
Depression			
Mild	3.632284	0.096	0.7941917—16.61247
Moderate	3.563075	0.036*	1.087312—11.67605
Severe	4.381086	0.046*	1.028044—18.67032
Extremely severe	3.448459	0.039*	1.063495—11.18187

CI: confidence interval; *significant

Sensitivity analysis confirmed the overall results for stress (p: 0.008) and depression (p: 0.003), and the results for different levels of stress (extremely severe levels of stress p = 0.005) and depression (p = 0.011 for moderate, p = 0.012 for severe, p = 0.014 for extremely severe).

Relapses were not influenced by smart-working (p = 0.966) or having at least one acquaintance diagnosed with COVID-19 (p = 0.999).

## Discussion

In our study, we found that there was a significant increase in the ARR in 2020, compared to 2019, with some patients experiencing two or three relapses/year, instead of one relapse for each patient in 2019.

Furthermore, we found an association between stress and depression and the risk of relapse in pwMS during the COVID-19 pandemic; in particular, between relapses and extremely severe levels of stress and between relapses and all levels of depression.

An association between stress and disease activity in MS has been assumed for a long time [18]; a meta-analysis reported a consistent association between stressful life events and subsequent exacerbation in MS [19].

Stress is also a potential trigger for radiological disease activity in MS; indeed, a significant relationship between moderately stressful life events and the appearance of new gadolinium-enhancing (Gd +) lesions 4 to 8 weeks later have been demonstrated [3].

The mechanisms by which stress might lead to MS disease activity are not clear; however, it has been hypothesized that it may be responsible for a disruption in the communication between the immune system and the two major systems involved in stress response: the hypothalamic–pituitary–adrenal axis and the autonomic nervous system. Insensitivity to glucocorticoid and beta-adrenergic modulation activated by stress might be involved in overshooting inflammation in MS [20]; in particular, neuropeptides secreted under stress, such as corticotropin-releasing hormone and neurotensin, activate microglia and mast cells to release inflammatory molecules. These lead to maturation and activation of T-17 autoimmune cells, disruption of the blood–brain barrier, and T cell entry into the central nervous system, thus promoting brain inflammation in MS [21].

In major depressive disorder in people without MS, feedback inhibition of ACTH secretion by cortisol is compromised independently of an age effect on the hypothalamic–pituitary–adrenal axis function [22]; in MS, an association among mood disorders, dysfunction of the hypothalamic–pituitary–adrenal axis and cerebral inflammation (cerebrospinal fluid white blood cell counts and presence of Gd + lesions on MRI) have been reported, too [23].

Furthermore, in RR-MS depression is related to the production of the pro-inflammatory cytokine IFN-gamma by autoaggressive T cells that might be decreased by treatment of depression [24]. Moreover, the cytokine profile of depressed MS patients is characterized by increased levels of proinflammatory cytokines, including TNF-α, IL-1β, and IL-6 in both cerebrospinal fluid and peripheral blood; furthermore, increased levels of IL-6 and decreased levels of IL-4 have been reported in depressed MS patients when compared to non-depressed MS patients and healthy controls and CD8 + T lymphocyte levels are significantly increased in MS depressed versus non-depressed patients [25]. Altogether, these immune changes associated with stress and depression may play an important role in triggering MS relapses.

Moreover, in our study, anxiety and PTSD were not associated with relapse; however, unlike depression, to date controversies still exist regarding the impact of anxiety on the human immune system.

Most studies on the adaptive immune role in anxiety have examined the presence of CD4 + (T helper) and CD8 + (cytotoxic) T lymphocytes, immune cells known to be involved in the pathogenesis of MS. One study that examined a mixed group of patients with either generalized anxiety disorder or panic disorder showed a lower CD4 + /CD8 + T cell ratio in blood leukocytes compared to healthy volunteers, which was due to an absolute increase in the number of CD8 + T cells in patients [26]. Conversely, a separate study of only patients with panic disorder showed decreased CD8 + T cells and a related increase in CD4 + /CD8 + T cell ratio [27]. On the other hand, in individuals with PTSD, one study showed significantly reduced overall lymphocyte counts, which reflected lower absolute numbers of CD4 + and CD8 + T cells compared to individuals without PTSD [28]. Moreover, in a separate study, women with PTSD had an altered T cell profile with increased CD4 + T cells and decreased CD8 + T cells [29]. Across the entire sample in this study, there was a strong correlation between CD4 + /CD8 + T cell ratio and the presence of PTSD symptoms [29].

Overall, these studies, on small numbers of subjects, show alterations in adaptive immune profiles in anxiety disorders, and yet the composition of immune cells in blood samples may be influenced by gender, age, and other humoral factors; these factors might contribute to the conflicting results across studies but they also show that immune changes related to anxiety are not necessarily shifted toward an immune profile known to be associated with relapses in MS. Hence, to better understand the role of anxiety on relapses, if any, it will be important to study lymphocyte subsets changes in pwMS with anxiety disorders, accounting for confounding factors. Another possible explanation for our negative results may be that questionnaires used to evaluate anxiety, PTSD and stress may detect different aspects of anxiety having different effects on the immune system and, hence in triggering relapses.

On the other hand, association does not imply causation; since the COVID-19 pandemic dramatically altered the standard of care in clinical practice [10], especially causing under-monitoring, the hampered management of pwMS might have limited the early identification of treatment failure, adverse events, or disease progression and thus therapy switch. In this scenario, we cannot exclude that in 2020, the increased relapse rate in our sample, might have been the consequence of under-monitoring with less and delayed therapeutic intervention (i.e., escalation) as a consequence of both less consultations and MRIs during the peak of the first wave (Mar 2020). Therefore, less and delayed therapeutic intervention might have caused relapses and worse psychological health.

## Limitations

A major limitation of our study is the lack of previous (in 2019) evaluation of depression, anxiety, and stress to establish whether stress and depression per se might have played a role in the occurrence of relapses or whether the prolonged stress and reactive depression to the pandemic played a major role.

However, comparing the distribution of relapses in 2019 and 2020, the increase in relapses was mainly in the second and third quarters of 2020, in the months following the declaration of the pandemic, suggesting a significant role of the pandemic in worsening the disease management and in inducing or increasing stress and depression and though in the occurrence of relapses in 2020.

Unfortunately, due to the pandemic restrictions, many pwMS stopped regular MRI monitoring, and we could not acquire information about the infra-clinical disease activity.

On the other hand, a relevant role played by the impaired management of pwMS provoked by the pandemic cannot be excluded so that a worse disease control could have led to an increase in stress and depression.

## Conclusion

In conclusion, our results further support the findings that major negative stressful events might be associated with clinical exacerbations [2]. Since the management of depression and stress has been proved to be useful to ameliorate the associated neuro-inflammatory state, in the particular context of the ongoing pandemic, neurologists should be encouraged to evaluate their patients also for mood disorders and levels of stress to early identify a clinical condition that may potentially trigger disease activity.
